# Mercury Exposure Assessment in Mother–Infant Pairs from Continental and Coastal Croatia

**DOI:** 10.3390/biom10060821

**Published:** 2020-05-27

**Authors:** Ankica Sekovanić, Martina Piasek, Tatjana Orct, Antonija Sulimanec Grgec, Marijana Matek Sarić, Sandra Stasenko, Jasna Jurasović

**Affiliations:** 1 Institute for Medical Research and Occupational Health, 10000 Zagreb, Croatia; asekovanic@imi.hr (A.S.); torct@imi.hr (T.O.); asulimanec@imi.hr (A.S.G.); jurasovic@imi.hr (J.J.); 2 Department for Health Studies, University of Zadar, 23000 Zadar, Croatia; msaric@unizd.hr; 3 Clinical Department of Obstetrics and Gynecology, Merkur University Hospital, 10000 Zagreb, Croatia; sandra.stasenko@zg.ht.hr

**Keywords:** fish consumption, dental amalgams, mercury, selenium, postpartum women

## Abstract

The main source of mercury (Hg) exposure in the general population is fish. Another possible source is dental amalgam. Here, we compare the levels of Hg and selenium (Se) in samples of maternal and fetal origin collected shortly after childbirth of healthy postpartum women in the coastal (*n* = 96) and continental (*n* = 185) areas of Croatia related to maternal seafood/fish consumption. We also evaluated Hg concentrations and maternal serum metallothionein (MT2) concentrations in relation to the number of dental amalgam fillings, and *MT2A*-5A/G (rs28366003) polymorphism. The levels of Hg and Se in maternal hair and blood/serum, placenta and cord blood/serum increased in relation to increasing fish consumption with the highest values in subjects from the coast. The concentrations of each element and between elements correlated across the matrices. Increasing amalgam number correlated linearly with increased Hg levels in maternal and cord serum and was not associated with serum MT2. No association of *MT2A*-5A/G polymorphism and Hg or Se levels were found. The results confirmed higher fish consumption in coastal vs. continental Croatia and increases of both Hg and Se related to fish consumption in all analyzed samples. Increased blood Hg reflected the predominant MeHg share from seafood, while increased serum Hg matched exposure from dental amalgams.

## 1. Introduction

Mercury (Hg) is a pervasive pollutant and toxicant of major concern for humans and wildlife. It is released in the environment via both natural and anthropogenic sources, and primary anthropogenic emissions (without re-emission processes) greatly exceed natural geogenic sources [1,2,3,4,5,6,7,8,9,10,11]. Mercury toxicity remains a global health concern, especially in mother–infant pairs as a vulnerable population group for the toxic effects of Hg, particularly as a risk for neurodevelopmental disabilities. Therefore, appropriate biomarkers of Hg exposure and its perinatal effects are continuously being sought [12,13,14,15,16,17].

Virtually everyone is exposed to Hg and its different chemical forms. Three main forms and sources of public concern are: exposure to MeHg by ingestion of fish, mercury vapor (Hg^0^) absorbed by inhalation after release from dental amalgam fillings, and exposure to ethylmercury through thiomersal-containing vaccines [3,4,5,16,18]. The general population is exposed to Hg mainly by seafood consumption, including fish, which is a source of highly toxic MeHg that bioaccumulates in marine organisms during the biogeochemical cycle of Hg. However, fish is also a highly nutritional staple food and a rich source of the essential ultra-trace element selenium (Se) [8,19,20,21,22]. A fish-rich diet is part of the cultural tradition in coastal areas of Europe, including the Mediterranean region and the Croatian Adriatic coast and islands, particularly in comparison to inland areas [23]. Similar geographic differences in seafood (fish) consumption are also known in other parts of the world, such as the United States, where the highest fish consumption was confirmed in coastal areas and the Northeast census region [24].

Another important source of inorganic Hg exposure in the general population is dental amalgam, which contains about 50% (43–54%) Hg^0^, 35% silver, 9% tin, 6% copper, and trace amounts of zinc. The rate of released Hg^0^ depends on the number, surface area, and chemical characteristics of amalgam fillings, personal eating habits, food texture, and personal physiological factors [5,18].

A major factor underlying the biochemical properties of Hg and its compounds, and causing their biological effects and interactions in the body, is the great predilection of ionic species of Hg to bond to reduced sulfur atoms, especially on small endogenous molecules containing a thiol (sulfhydryl, –SH) group, such as glutathione, homocysteine, metallothionein (MT), and also albumin. Its binding affinity to thiol-containing pharmacological agents is used for chelation therapy to remove Hg from endogenous ligands by forming new –SH-Hg complexes. Different chemical forms of Hg also have different toxicokinetics in the body, all of which induce toxic effects, depending on the form of Hg present at the time of exposure, as well as the duration, level, and route of exposure. The primary target for toxicity of inorganic Hg is the kidney where it is taken up, accumulated, and its toxicity expressed. Although organic Hg compounds can also have nephrotoxic effects, they have more diffuse systemic distribution and affect other organs, including the nervous, hematopoietic, cardiovascular, immune, respiratory, gastrointestinal, and reproductive systems [1,2,14,18,25,26]. The developing central nervous system is of the highest concern due to pronounced sensitivity to the toxic effects of environmental pollutants including Hg. As a chemical with the potential to cause irreversible neurodevelopmental disabilities, including autism and other cognitive impairments, Hg is a matter of particular public health concern [6,27,28].

During pregnancy, maternal Hg is partially accumulated in the placenta, and MeHg and Hg^0^ are predominantly transferred to the fetus who may already be affected in utero [28,29,30,31,32,33]. When assessing exposure and Hg disposition across the maternal-placental-fetal unit, it is best to concomitantly analyze maternal blood/serum, placenta and cord blood/serum together with the data on exposure sources. Whenever possible, epigenetic markers and specific gene polymorphism(s) in candidate genes should be determined, as they may modify the relationships of environmental exposure to Hg and susceptibility of the host [34,35,36].

More epidemiological studies are needed to assess special features of Hg exposure during pregnancy, especially by fish consumption, and to determine the potentially protective effects of nutrients closely interrelated with MeHg, such as Se, together with individual genetic susceptibility to Hg toxicity. The aim of this study, conducted as part of a broader cross-sectional population study in healthy postpartum women in Croatia, is to determine Hg and Se concentrations in samples of maternal and fetal origin (maternal hair and blood/serum, placenta and cord blood/serum) collected shortly after childbirth, and to compare these biomarkers between study participants from the coastal and continental areas with presumed different seafood/fish consumption and related Hg exposure. Irrespective of the geographic area of residence, we evaluated Hg concentrations in relation to the number of dental amalgam fillings as an additional source of Hg exposure, maternal serum metallothionein (MT2) concentrations (in coastal area subjects only) and *MT2A*-5A/G (rs28366003) polymorphism.

## 2. Materials and Methods

### 2.1. Recruitment of the Study Participants

**Figure 1:**
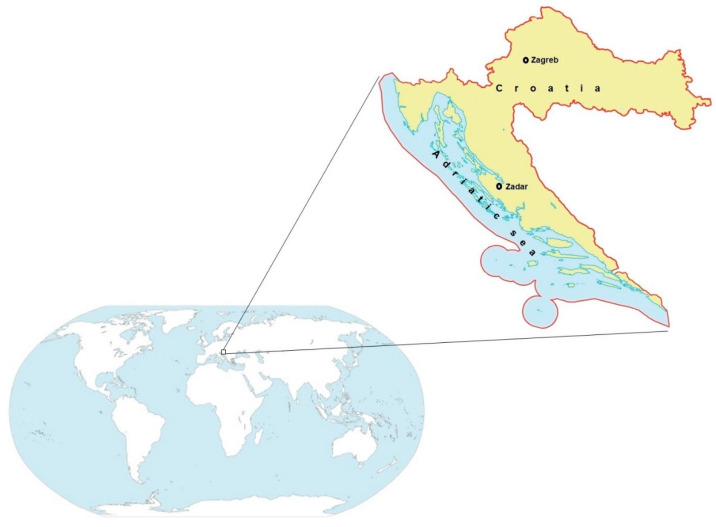
Location of the residence areas of recruited mother–infant pairs in Croatia.

This study was carried out using personal data and selected biomarkers obtained in two cohorts of mother–infant pairs recruited from two geographic areas of residence in Croatia (Figure 1): continental—the Zagreb metropolitan area, located in the northwest inland area (*n* = 185 participants) and coastal—the city of Zadar and its surroundings in Zadar County located on the eastern Adriatic coast (*n* = 96 participants). Inclusion criteria for this study, as part of a wider study conducted within a national research project, were a term vaginal delivery (between 37 and 42 weeks of pregnancy) in the maternity ward and absence of any chronic illness, major complication during pregnancy or twin pregnancy. Study participants fulfilling these criteria were informed about the research scope, methods, and biological samples to be collected and included in the study after the endorsement of informed consent forms. The research protocol for studying exposure to the main toxic metals, cadmium (Cd), lead (Pb), and Hg, and their effects in pregnancy and postnatal period within the entire research project was reviewed and approved by the ethics committees of three collaborating institutions (between 2006 and 2008, prior to commencement of the study): Merkur University Hospital in Zagreb, Zadar County General Hospital, and the Institute for Medical Research and Occupational Health in Zagreb where the study was conducted. The ethical approvals and consent of study participants are still valid and encompass all future analyses. Accordingly, in 2015 an addendum to the previous ethical approval was obtained from the Institute for Medical Research and Occupational Health in Zagreb prior to starting additional analyses of the specific single nucleotide gene polymorphism and metallothionein (MT2) concentrations in previously collected biological samples of maternal origin within this research project.

Data on sociodemographic characteristics, medical history, mineral-vitamin supplement intake, number of amalgam fillings, and dietary habits of postpartum women focused on seafood consumption, were collected by a questionnaire as described elsewhere [37,38] and from clinical records. The questionnaire was filled in with the help of an investigator in the research project or the appointed collaborator (medical health professional) employed in the maternity ward of the participating hospital. In this study, we used data collected by our previously created (not standardized) questionnaire that included the following data: general personal data (age and place of birth); professional history (education, position, and current employment); data on potential recent environmental exposure (place of residence, moving in the last 24 months, vicinity of factory or smelter, vicinity of highway/street/road/large cross-roads or bus terminal with heavy traffic); general health status (height, body weight at term, average weight before pregnancy, and body weight gain during pregnancy); data on the last pregnancy and delivery (number of previous pregnancies—parity, week of pregnancy at delivery, specific health problems during pregnancy, such as increased blood pressure and blood glucose, peripheral edema or other major health problems, number of previous abortions or miscarriages and twins); data on the newborn (sex, weight, length at birth, and APGAR score at birth in 1^st^ and 5^th^ min, a vitality index (named after its creator in 1952, an anesthesiologist Virginia Apgar) that provides an accepted and convenient method for reporting the clinical status of the newborn infant immediately after birth based on a rating of 0, 1, or 2 for each of the five characteristics: A—appearance (skin color), P—pulse (heart rate), G—grimace (response to stimulation of the sole of the foot), A—activity (muscle tone), and R—respiration (respiratory effort), with 10 being a perfect score); maternal dietary history (self-identification of the predominate diet type: mixed, vegetarian, ovo-vegetarian, lacto-ovo-vegetarian, frequency of consuming fish, shellfish, tinned fish per week or month); smoking habit (self-classification as active, passive or previous smoker; for active smokers: number of cigarettes or packages per diem, age when started, smoking during last pregnancy; for non-smokers: data on potential passive smoking; data on active smokers in the family and/or at workplace with duration of exposure in hours to passive smoking per diem); and number of dental amalgam fillings (irrespective of amalgam surface). For the purpose of this study, we focused on the data on the self-reported frequency of seafood/fish consumption (expressed per week) and number of amalgam fillings as the main sources of Hg exposure, and the related parameters.

Based on self-reported data on seafood consumption, including fresh/frozen and tinned fish and shellfish, subjects were categorized into four subgroups: 0—no consumption; 1—consumption ≤1 per week; 2—consumption >1–2 per week; 3—consumption >2 per week. Based on the number of amalgam fillings, participants were categorized into four subgroups: 0—no amalgam fillings; 1–2—one or two amalgam fillings; 3–4—three or four amalgams fillings; ≥5—five or more amalgam fillings. According to MT2A-5A/G polymorphism, the participants were assigned into two subgroups: wild-type (AA genotype) and G allele carriers (AG/GG genotype).

### 2.2. Collection and Preparation of the Samples and Element Analysis

Biological samples collected in the period between 2008 and 2010 in maternity wards included: maternal blood and hair, placenta, and umbilical cord blood. Immediately after childbirth, maternal and cord blood were sampled in two vacutainer tubes (BD Vacutainer^®^ Trace Element, Becton Dickinson, New Jersey, NJ, USA) with K_2_-EDTA anticoagulant (4 mL) and without anticoagulant (6 mL). Whole placentas were placed in marked zip-lock polyethylene bags. Hair samples were collected by cutting with clean scissors from the nape of the neck as close as possible to the root. Blood serum was separated by centrifugation at +4 °C and 3000 rpm for 20 min (by Hettich Rotanta/R, type 3501, Hettich-Zentrifugen, Tuttlingen, Denmark) and decanted into clean polyethylene tubes. All collected samples were transported in cooler bags to the laboratories of the Institute of Medical Research and Occupational Health in Zagreb where placentas were sampled, as described earlier [37,38], and all samples of placenta, whole blood, and serum were stored in the freezer at −20 °C until analysis. Hair samples were stored in paper envelopes at room temperature.

Blood and serum samples were diluted before analysis with a solution containing 0.7 mM NH_3_, 0.01 mM EDTA, and 0.07% (v/v) TX-100 [39]. Dilution factors for blood and serum were 80 and 20. Placental samples were digested with purified concentrated nitric acid and ultrapure water (2:1) in a microwave digestion system UltraCLAVE IV (Milestone, Sorisole, Italy) according to the program outlined in the Supplementary Materials (Table S1). The sub-boiling distillation duoPUR system (Milestone) was used to purify nitric acid (65% p. a., Merck, Darmstadt, Germany). After digestion, samples were adjusted to 8 g with ultrapure water (GenPure, TKA System GmbH, Niederelbert, Germany), stored at +4 °C, and additionally diluted 20-fold before analysis.

The number of hair samples available for element analysis was lower (*n* = 71) than originally collected from the participants of the continental area because part of the samples was used for nicotine analysis related to maternal cigarette smoking and its association with Cd exposure biomarkers [40]. Each hair sample was cut to a length of 3 cm from the root, as this approximate length corresponds to the third trimester of pregnancy and correlates with Hg in cord blood [41,42]. Samples (weighed 0.05–0.1 g) were placed into quartz tubes, washed by ultrasonic agitation in acetone for 10 min, soaked in 0.2% TX-100 for 10 min, rinsed 3–4 times with ultrapure water, and ultimately soaked in acetone for 10 min and dried at 75 °C for 3 h [43]. Washed hair samples were weighed, digested with 4 mL of purified concentrated nitric acid and ultrapure water (1:1) in UltraCLAVE IV according to the conditions presented in Table S1, adjusted with ultrapure water to 6 g, stored at +4 °C, and diluted 4-fold before element analysis.

Concentrations of Hg and Se in maternal hair, blood and serum, placenta and cord blood, and serum were determined by inductively coupled plasma–mass spectrometer (ICP-MS) Agilent 7500cx (Agilent Technologies, Tokyo, Japan). The ICP-MS optimized working conditions are shown in Table S2. Concentrations of Hg and Se in blood and serum samples were quantified by matrix matched calibration. Calibration curve standards prepared in 1% HNO_3_ were used for quantification of digested samples of hair and placenta. The samples were prepared and analyzed in a laboratory with a heating, ventilating, and air conditioning (HVAC) system combined with high efficiency particulate air (HEPA) filters.

The accuracy of measurements was checked by commercial available reference materials: ClinChek^®^ Whole Blood Controls (Level I, II, and III) and Serum Controls (Level I and II) (Recipe, Munich, Germany), Seronorm^TM^ Trace Elements Whole Blood (Level I and II) and Serum (Level I and II) (Sero AS, Billingstad, Norway), bovine liver BCR 185R, pig kidney BCR 186R, mussel tissue BCR 278R (IRMM, Geel, Belgium), human hair IAEA-086 (IAEA, Vienna, Austria), and human hair CRM 13 (NIES, Tsukuba, Japan) (details provided in Table S3).

### 2.3. Genotyping of MT2A-5A/G Polymorphism

DNA was isolated from maternal blood using the QIAamp DNA Blood Mini Kit (Qiagen, Hilden, Germany), according to the method provided by the manufacturer, and stored at −20 °C until used. BioSpec-nano (Shimadzu, Kyoto, Japan) was used to check the concentration and purity of the isolated DNA. To amplify the signal of the fragment 241-bp polymerase chain reaction (PCR), GeneAmp PCR System 2700 (Applied Biosistems, Waltham, MA, USA) was used with the following primers:

forward: 5´- CGC CTG GAG CCG CAA GTG AC;
reverse: 5´- TGG GCA TCC CCA GCC TCT TA.

Single nucleotide polymorphism (SNP) of *MT2A*-5A/G was genotyped by the method of restriction fragment length polymorphism (RFLP) by incubation of the PCR products at 37 °C for 1 h and 45 min with *BsgI* restriction enzyme (New England BioLabs, Hitchin, UK) as described in our previously published paper [44]. Digested samples were separated by electrophoresis on 2% agarose gel with ethidium bromide and visualized under an ultraviolet illuminator. The presence of 144-bp, 56-bp, and 41-bp fragments were characteristic for the A allele, whereas 185-bp and 56-bp fragments defined the G allele. The results of RFLP were confirmed by repeating the analysis in 30 randomly selected samples.

### 2.4. Metallothionein (MT2) Analysis in Maternal Serum

The levels of metallothionein (MT2) in serum were determined in a part of the study participants (only subjects from the coast, *n* = 94) using enzyme-linked immunosorbent assay (ELISA) kit SEB868Hu and the double-antibody sandwich method as recommended by the manufacturer (Cloud-Clone Corp, Katy, TX, USA). TECAN Infinite F50 (TECAN Group Ltd., Männedorf, Switzerland) was used to measure MT2 absorbance at 450 nm.

### 2.5. Statistical Analysis

Data were expressed as the median (25–75% interquartile range) and/or mean with standard deviation (SD). The values of elements’ concentration below the limit of detection (LOD) were substituted with the LOD/2 value. Statistical significance was set at 5% (*p* < 0.05). The Kolmogorov–Smirnov test was used to test the normality of variables and logarithmic (ln) transformation was used to normalize variables before statistical analysis. Differences between the groups were tested by Mann–Whitney U test or by one-way ANOVA with post hoc Bonferroni test in the case of three or more subgroups (based on the frequency of seafood consumption and the number of amalgam fillings). The relationships between Hg and Se in all measured samples, maternal seafood consumption, amalgam fillings, and maternal serum MT2 were analyzed by Spearman’s rank correlation. Forward stepwise multiple linear regression analysis was used to evaluate the effects of maternal seafood consumption and amalgam fillings on Hg levels in maternal blood, serum and hair, placenta and cord blood, and serum. The independent variables included in the regression models were residence area, seafood consumption per week, maternal amalgam fillings (the four-level variable was transformed to three new dummy coded dichotomous variables, using zero amalgam fillings as the reference category), maternal age, education, parity, and maternal weight gain during pregnancy. For the final model, only variables at *p* < 0.05 were included. The software package TIBCO Statistica™, version 13.5.0.17 (TIBCO Software, Inc., Palo Alto, CA, USA) was used for statistical analysis and GraphPad Prism 7 (GraphPad Software, Inc., San Diego, CA, USA) for graphic illustration of results.

## 3. Results

Table 1 shows the general characteristics of the participants included in this study, based on the data collected in the questionnaire. General maternal data, seafood intake, and data on newborns and placenta are presented for the cohorts from the continental (*n* = 185) and coastal area (*n* = 96), with the available data on SNP MT2A-5A/G frequency (*n* = 268) and the number of dental amalgams (*n* = 208) in participants from both study areas. Average maternal age was 29 years. The study participants matched in parity, body weight and height before pregnancy, average weight gained during pregnancy, gestation week at delivery, birth weight and length, median APGAR vitality scores in the 1^st^ and 5^th^ min, and trimmed fresh placental weight. Regarding seafood consumption, most self-declared data actually referred to fresh and frozen marine fish, and only partially to tinned fish. There were marked differences between the studied cohorts; in the continental area 54% of subjects self-reported that they ate fish less than once a week and 11% ate no fish at all, whereas among subjects from the coastal area more than 50% of participants reported fish consumption at least once or more than twice a week. With regard to determined genotype frequencies of MT2A-5A/G polymorphism, among 268 participants, 94.4% subjects were typical homozygote (AA) and 5.6% subjects were G-allele carriers. Among the latter, 5.2% subjects were heterozygote (AG) and 0.4% atypical homozygote (GG). The allele frequencies were in accordance with the Hardy–Weinberg equilibrium (χ^2^ = 2.58, *p* = 0.108). Regarding the presence of dental amalgam fillings, 18% of participants had no dental amalgams and 82% of participants had at least one amalgam filling; specifically, 30% had one to two, 22% had three to four, and 30% had five or more dental amalgams.

Significantly higher concentrations of both Hg and Se were found in all measured samples of the maternal-placental-fetal unit in the coastal vs. continental areas. These increases in Hg concentrations were 2.3 to 3.5-fold, while the ratio between Se concentrations in the two study groups was up to 1.2 (Table 2).

**Figure 2:**
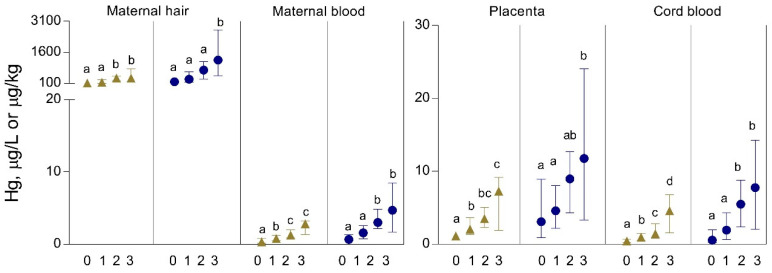
Concentrations of Hg in maternal hair and blood, placenta, and cord blood in study participants from Croatia, continental (▲) and coastal area (●), related to the frequency of seafood (fish) consumption per week. Subgroups (number of participants is given in Table 1, except the number of analyzed hair samples in the continental area indicated here in parentheses): 0—no consumption (*n* = 11); 1—consumption ≤1 per week (*n* = 34); 2—consumption >1–2 per week (*n* = 20); 3—consumption >2 per week (*n* = 6). Data are presented as the median and interquartile range (25–75%). Statistically significant differences (*p* < 0.05) between the subgroups tested by one-way ANOVA and post hoc Bonferroni test are indicated by different letters.

The concentrations of Hg in maternal hair and blood, placenta, and cord blood in the study participants from the continental and coastal area with regard to the frequency of seafood (mainly fish) consumption per week based on self-reporting are presented in Figure 2.

In both geographic areas of residence, all Hg biomarkers increased with increasing maternal seafood consumption. In participants who consumed seafood at least once per week compared to those who ate no seafood, Hg levels were 1.5-fold higher in maternal hair, 2-fold in maternal blood, 1.5-fold in the placenta, and 2–3-fold in cord blood. In subjects who consumed seafood twice per week vs. subjects who ate no seafood, these differences were highly significant (*p* < 0.0001) with Hg levels that were higher 3–7-fold in maternal hair, 7–8-fold in maternal blood, 4–6-fold in the placenta, and >10-fold in cord blood.

**Figure 3:**
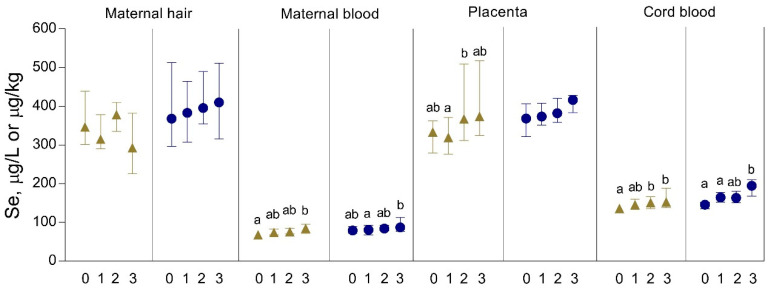
Concentrations of Se in maternal hair and blood, placenta, and cord blood in the study participants from Croatia, continental (▲) and coastal area (●), related to the frequency of seafood (fish) consumption per week. Subgroups (number of participants is given in Table 1, except the number of analyzed hair samples in the continental area indicated here in parentheses): 0—no consumption (*n* = 11); 1—consumption ≤1 per week (*n* = 34); 2—consumption >1–2 per week (*n* = 20); 3—consumption >2 per week (*n* = 6). Data are presented as the median and interquartile range (25–75%). Statistically significant differences (*p* < 0.05) between the subgroups tested by one-way ANOVA and post hoc Bonferroni test are indicated by different letters.

The concentrations of Se in maternal hair and blood, placenta, and cord blood in the study participants from the continental and coastal area with regard to the frequency of seafood (mainly fish) consumption per week based on self-reporting are presented in Figure 3. As for Hg concentrations, Se biomarkers in subjects from both geographic areas of residence increased with increasing maternal seafood consumption, though to a lesser extent, with the exception of hair Se concentrations. In subjects who consumed seafood more than twice per week vs. subjects who ate no seafood, the ratios in maternal and cord blood Se concentrations were up to 1.3.

**Figure 4:**
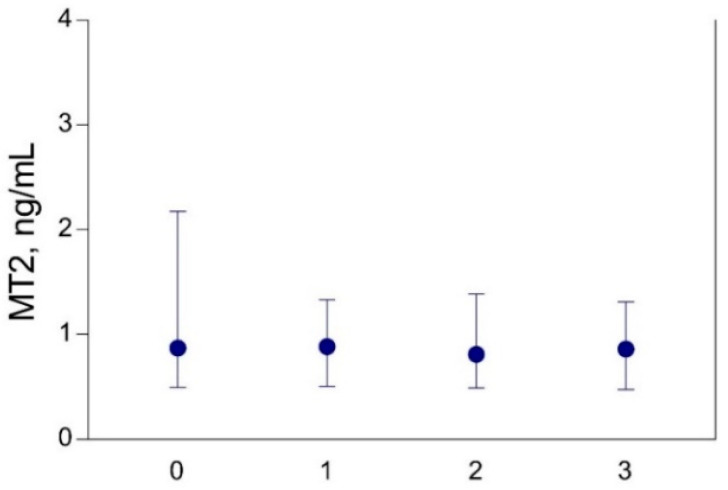
Concentrations of metallothionein (MT2) in maternal serum in a part of the study participants (only subjects from coastal Croatia, *n* = 94) related to the frequency of seafood (fish) consumption per week. Subgroups: 0—no consumption (*n* = 4); 1—consumption ≤1 per week (*n* = 38); 2—consumption >1–2 per week (*n* = 31); 3—consumption >2 per week (*n* = 21). Data are presented as the median and interquartile range (25–75%).

**Figure 5:**
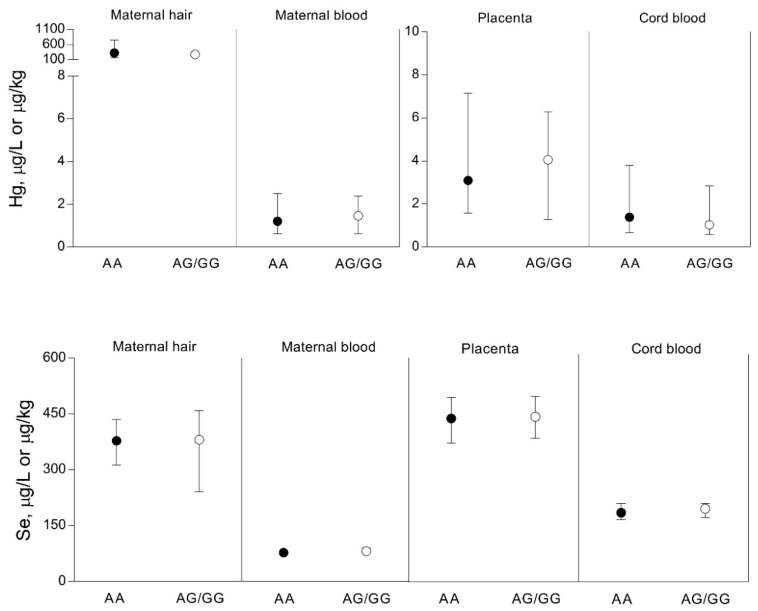
Concentrations of Hg and Se in maternal hair and blood, placenta, and umbilical cord blood in the study participants from Croatia (*n* = 268) related to *MT2A*-5A/G polymorphism. Subgroups are denoted by the genotype: AA: wild-type (*n* = 253); AG/GG: G allele carriers (*n* = 15). Data are presented as the median and interquartile range (25–75%).

In all study participants from both geographic areas, no differences were found in the concentrations of MT2 in maternal serum between the subgroups in relation to the frequency of seafood (fish) consumption (Figure 4). No differences were found in the concentrations of both Hg and Se in maternal hair and blood, placenta, and cord blood with regard to the MT2A-5A/G polymorphism (Figure 5). 

In subjects with dental amalgams (one or more) compared to subjects without dental amalgams (Table 3), Hg concentrations were 1.3-fold higher in maternal serum and 1.5-fold in cord serum, whereas levels of Hg in maternal hair and blood, placenta, and cord blood did not differ. 

**Figure 6:**
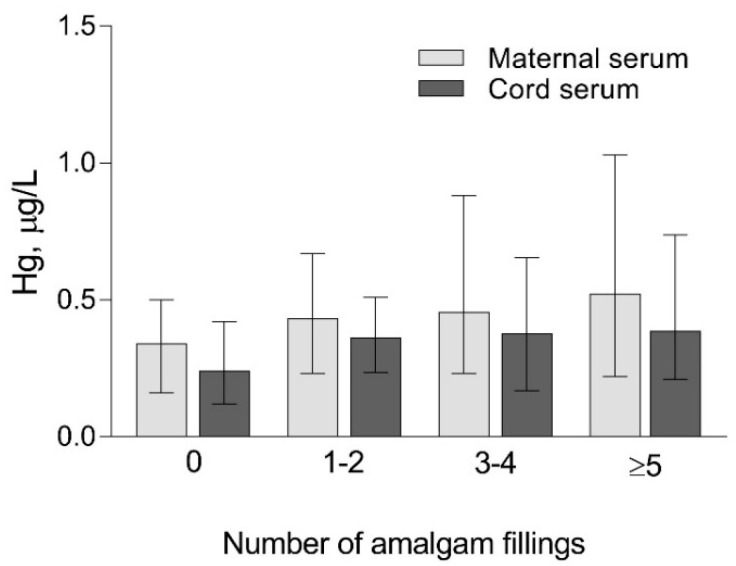
Concentrations of Hg in maternal and umbilical cord serum related to the number of amalgam fillings. Subgroups: 0—no amalgams (*n* = 38); 1–2—1 to 2 amalgams (*n* = 62); 3–4—3 to 4 amalgams (*n* = 46); ≥5—5 or more amalgams (*n* = 62). Data are presented as median and 25–75% interquartile range.

**Figure 7:**
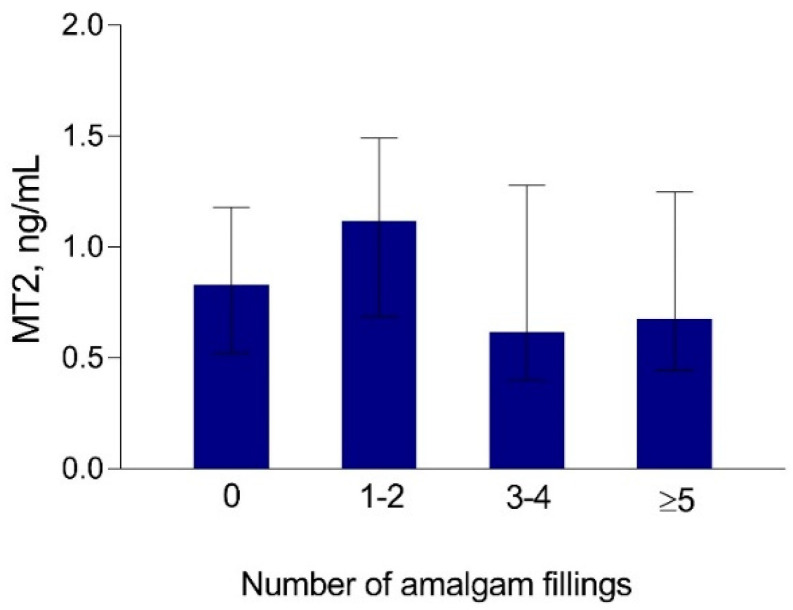
Concentrations of MT2 in maternal serum in the study participants from the coast (*n* = 94) related to the number of amalgam fillings. Subgroups: 0—no amalgams (*n* = 27); 1–2—1 to 2 amalgams (*n* = 29); 3–4—3 to 4 amalgams (*n* = 26); ≥5—5 or more amalgams (*n* = 12). Data are presented as median and 25–75% interquartile range.

We found no difference between the subgroups of study participants related to the number of dental amalgams in either concentrations of Hg in maternal and cord serum (Figure 6) or MT2 concentrations in maternal serum (Figure 7).

Spearman’s correlation coefficients (ρ_s_) between maternal age, seafood (fish) consumption, number of amalgam fillings, maternal serum MT2, and the concentrations of Hg and Se in the measured samples are presented in Table 4. Maternal seafood intake positively correlated with virtually all the determined element biomarkers, except maternal hair Se. Good or strong correlations (ρ_s_ = 0.62–0.86) were found between Hg levels in all measured samples of maternal and fetal origin and for Se in maternal blood and serum (ρ_s_ = 0.8), whereas correlations between Se levels in other measured samples were weak (ρ_s_ = 0.18–0.55). In all analyzed samples, the levels of Hg and Se were significantly positively correlated (ρ_s_ = 0.19–0.52). MT2 concentration was inversely correlated with Se in maternal blood (ρ_s_ = −0.28), while maternal amalgam number (as represented in Table 1) was correlated with Hg in maternal and umbilical cord serum (ρ_s_ = 0.16). Maternal age correlated with seafood intake, Hg in maternal and umbilical cord blood and serum, Hg in placenta, and Se in maternal blood and serum (ρ_s_ = 0.12–0.2). These results, together with the significant correlations between Hg biomarkers and residence area, education, parity, and weight gain (results not shown), suggest a complex association among the variables. Each of these studied variables was therefore considered as potentially explanatory/confounding in their influence on Hg levels.

Table 5 shows the results of the forward stepwise multiple linear regression analyses to further evaluate the influence of maternal seafood consumption and the number of amalgam fillings on Hg concentrations in maternal hair, blood and serum, placenta, and umbilical cord blood and serum (using ln transformed data). Confounding variables included in the model were area of residence, maternal age, education, parity, and maternal weight gain during pregnancy. Seafood consumption (frequency of seafood consumption per week) and geographic area of residence of the study participants were significant predictors of increased Hg levels in all of the analyzed biological samples. The number of amalgam fillings was a significant predictor of increased Hg concentrations in maternal and cord serum and also in maternal blood, though to a lesser extent. There was a linear trend of increasing Hg levels in maternal and cord serum with the number of amalgam fillings.

## 4. Discussion

This paper presents data on Hg and Se levels measured in samples collected shortly after childbirth that represent concentrations in three compartments of the maternal-placental-fetal unit, comprising maternal hair, blood and serum, placental tissue, and umbilical cord blood and serum. The results were partly already published [44] and here they are scrutinized further to elucidate the impact of seafood consumption and amalgam fillings on Hg levels. We compared the cohorts of healthy postpartum women from two geographic areas of residence, the Zagreb metropolitan area as a typical inland area, and the city of Zadar and the surroundings in Zadar County of coastal Croatia, one of the most important landing places for small pelagic species and demersal catches on the Croatian Adriatic coast. Seafood/fish consumption was evaluated as the main source of Hg exposure. In addition, the relationship between maternal dental amalgams and Hg serum levels in maternal and cord blood was assessed irrespective of geographic area. Even though Croatia is a small country (56,594 km^2^ terrestrial and 31,710 km^2^ maritime area), it has a very diverse topography; from the Mediterranean to Central European, mountainous to flat, coastal to continental. It ranks as one of the top five European countries with regard to biodiversity [45]. There is also diversity in the regional cultural traditions, including the consumption of fish that is higher in coastal than in continental areas. However, total consumption of fish and fish products per capita in Croatia is generally significantly lower than in other Mediterranean countries [46].

### 4.1. Hg in Maternal Hair and Blood, Cord Blood and the Placenta

During its formation, hair sequesters MeHg by binding to –SH groups in keratin, and after exposure to MeHg, hair Hg consists of just over 80% MeHg. In group studies, hair specimens are regarded as accurate measures for the evaluation of exposure to Hg, especially in the assessment of MeHg exposure by fish consumption [5,14,16,47,48]. Inorganic Hg is poorly accumulated in hair and total Hg concentration in hair reflects MeHg exposure, even in persons who do not eat fish [49]. 

Our results confirm an association of seafood/fish consumption and Hg concentration in maternal hair [41,42,50,51]. Although the overall median Hg level in hair was 0.29 mg/kg (interquartile range 0.16–0.69 mg/kg), one participant (1.4%) in the continental and 25 participants (21%) in the coastal area had Hg levels in hair >1.2 mg/kg (i.e., 10% of the estimated benchmark dose level recommended by the National Research Council (NRC, USA) [28]). Participants residing in the coastal area had 2.3-fold higher hair Hg than in the continental area, which is in agreement with previous findings that showed significantly higher Hg levels in postpartum women from coastal than continental Croatia, with medians of 0.471 mg/kg vs. 0.129 mg/kg, respectively [51]. Maternal hair Hg levels in coastal Croatia were higher than in most Northern and Central European countries, reported in by the European Union (EU) study CHOPES/DEMOCHOPES to be 0.225 mg/kg; this is in line with the levels in pregnant women receiving a habitual diet in a randomized controlled trial in Norway (0.485 mg/kg), but lower than in Mediterranean countries, namely, Portugal, Spain, and Greece (>1 mg/kg) [50,52,53,54]. Recently reported total Hg levels in hair of 0.9 mg/kg in Portuguese postpartum women, of which 38% exceeded the safety limits established by the United States Environmental Protection Agency (US EPA), had no association with birth weight or length [55].

In comparison with population groups in North America having traditionally high seafood/fish intake, participants from the coastal area in this study who consumed seafood more than twice per week had three-fold lower Hg levels in hair compared to Hg levels in the maternal hair of, for example, Northern Québec Inuit postpartum women in the third trimester [41]. However, in a prospective cohort study evaluating the costs and benefits of fish consumption during pregnancy for brain development carried out in eastern Massachusetts, a part of the United States with known increased fish consumption [24], the mean Hg levels in maternal hair were 0.55 mg/kg (0.02–2.38 mg/kg) and study participants consumed on average 1.2 fish meals per week, similar to the participants in this study. It was concluded that a higher fish intake in pregnancy is associated with better cognitive score in the offspring and higher Hg levels are associated with lower infant cognition, indicating that during pregnancy women should eat fish, though preferably varieties with a lower Hg content [56]. In accordance with these results is an interesting finding reported in a preliminary study in Croatia showing significantly smaller infant cerebellum length if Hg levels in maternal hair exceeded levels of 1 mg/kg [57].

As in hair, MeHg in blood is also the main contributor to total Hg levels and the concentrations of Hg in blood show a direct relationship with hair Hg levels. The typical hair-to-blood ratio is estimated at 250, corresponding to the overall average of values for individuals reported in published studies from 140–370, although large variations exist, especially in persons with infrequent fish consumption [1,5,8,12,16,28]. In our study participants, the ratios between Hg concentrations in hair and maternal blood were 208 in the continental and 188 in the coastal area. For exposure assessment, it is best to obtain both biomarkers, hair Hg and blood Hg (including cord blood), which provide accurate and reliable biomarkers of MeHg exposure, along with dietary information on fish consumption and other food [58]. Total Hg concentrations in the measured matrices showed differences between the studied cohorts as seen in a previous study in Croatia for Hg in maternal hair and cord blood [51]. Blood Hg levels in participants residing in the coastal area were similar to previously published values in mother–infant pairs from Croatia, with a reported concentration in maternal blood of 2 µg/kg and in cord blood of 2.9 µg/L [52], 2.9 µg/kg [51], and 3.41 µg/kg [59]. The values were also similar to those reported for mother–infant pairs in the Italian part of the Adriatic coastal area (i.e., 2.4 µg/kg in maternal blood and 3.9 µg/kg in cord blood [52]), but lower than values reported in other Mediterranean countries such as Spain and Greece [60,61]. At the same time, maternal and fetal Hg levels in participants from continental Croatia were similar to the values of 0.661 µg/L in cord blood reported previously for the Croatian population [51], and also the values of maternal blood and cord blood in Central European countries: 0.7 µg/L and 1.1 µg/L in Austria [62] and 0.63 µg/L and 0.8 µg/L in Slovakia [63]. The differences found between geographic areas in Croatia in this study are due to the higher frequency of fish consumption along the coast; data indicate that more than 55% of subjects in the coastal area reported fish consumption at least once a week, as opposed to 55% of subjects in the continental area who reported fish consumption of less than once a week or never.

Exposure to MeHg during prenatal life, especially during critical periods of central nervous system development, may cause serious and irreversible impairments [64]. Organic forms of Hg easily pass the placenta, most likely via neutral amino acid carriers [65]. Hg levels in umbilical cord blood may be 20–65% higher than the levels in maternal blood [32]. A similar transport mechanism of MeHg through the blood–brain barrier includes MeHg-cysteine conjugate and methionine [66]. Concentration of Hg in cord blood better reflects fetal exposure than Hg in maternal hair [67]. We found differences (*p* < 0.02) in the ratios of Hg concentrations in cord blood to maternal blood that were 1.6 in coastal and 1.2 in continental mother–infant pairs. Based on the meta-analysis, the ratios between cord and maternal blood concentrations of MeHg and inorganic Hg are estimated at 1.89 and 1.01 [68]; hence comparing the ratios for total Hg concentrations in our study, we can assume that there were differences in exposure sources to Hg between the two studied areas. With regard to the levels of concern accepted as the current US EPA reference dose (RfD) for concentrations of total Hg of 6.4 μg/L and the equivalent for MeHg of 5.8 μg/L [24,28,58], we determined these Hg levels in blood in 10% of postpartum women and 31% of infants (cord blood) in the coastal area, compared to only <1% postpartum women and 3% infants in the continental area. The ratio of Hg concentrations in cord and maternal blood was reportedly higher for infants in Croatia and Slovenia [52,69]; however, our ratio results are lower than in Spain or other high-fish consuming countries, where >60% of Hg levels in infants exceeded the RfD value (see [70]). Studies conducted in Spain showed that 90% of pregnant women consumed seafood/fish ≥3 times per week, leading to high prenatal and early childhood exposure to Hg and MeHg measured in cord blood and hair samples of infants and preschool children [70,71]. In our study, only 7% of participants consumed seafood ≥3 times per week and we identified a gradual increase in Hg concentrations in maternal and cord blood and placenta related to increased seafood consumption.

The strong positive correlation (ρ > 0.8) between Hg levels in maternal blood and cord blood is in line with previous findings [29,63,72]. Furthermore, we found a positive correlation between Hg levels and maternal age and education level, which is in agreement with several reports on the association of fish consumption and age, level of education, and household income [24,55,73,74,75]. It is likely that persons with higher education levels more frequently consume seafood/fish as they are familiar with the health benefits of such a diet as a rich source of essential nutrients.

The literature data on Hg concentrations in the placenta are scarcer than on the concentrations of two other main toxic metals, Cd and Pb. The reported Hg levels in the placenta under conditions of low to moderate environmental exposure vary from 2 to 13 μg/kg with an average of 8 μg Hg/kg, based on wet weight [30,31]. In this study, placental Hg levels were within this range with values about three-fold higher in the coastal than in the continental areas of residence. Based on associations of seafood intake frequency and Hg concentrations in the placenta, placental Hg levels can be considered to be a valuable biomarker of exposure to Hg from moderate seafood consumption. A Portuguese study reported the highest levels of Hg in the amniotic membrane, whereas Hg levels were similar in the decidua basalis, chorionic plate, and umbilical cord, and the authors recommended further studies on a larger sample size to better understood the role of the amniotic membrane in placental–fetal Hg accumulation [55].

### 4.2. Hg and Dental Amalgams

Amalgam fillings have been used for dental restoration for over 150 years and they are still used in many countries, including Croatia, due to their advantages, such as simple preparation, hardness, effectiveness, duration, and low cost. Apart from an unattractive appearance, the major disadvantage of dental amalgam is the potential risk of exposure to Hg^0^ released into the mouth and saliva during chewing and placement and/or removal. The estimated daily absorbance of Hg from amalgam fillings ranges from 3–17 µg [13], whereas daily absorbance from all forms of Hg from seafood/fish is estimated at 2.31 µg, and about 0.3 µg Hg from other foods, air, and water [1]. The rate of released Hg^0^ depends on the number, surface areas, and chemical characteristics of the amalgam filling(s), personal eating habits, food texture, and personal physiological factors. To this end, amalgam fillings have been the subject of an ongoing dispute for centuries, known as “amalgam wars”, due to concern of health effects of released Hg [1,4,5,13,76,77,78,79,80,81,82]. The number of amalgam fillings is associated with Hg concentrations in the adult brain and urine, and has a tendency to be associated with Hg in the fetal kidney (but not in the fetal brain), amniotic fluid, cord blood/serum, and/or placenta [29,32,63,83,84,85,86], though no associations have been found with fetal biometric measurements [77,78,87]. A recent Avon Longitudinal Study of Parents and Children (ALSPAC) study reported that women with four or more dental amalgams at the beginning of pregnancy had higher Hg blood levels [88]. Although the European Food Safety Authority (EFSA) reported that the tolerable weekly intake for inorganic Hg might be exceeded due to the additional inhalation exposure in people with a large number of amalgam fillings, the evidence is weak, and the data are mainly derived from model-based calculations [8]. Our results of higher Hg levels in maternal and cord serum in participants with at least one dental amalgam filling compared to those with no amalgams are in agreement with findings in Swedish adolescents showing that dental amalgam is one of the determinants (in the multiple regression analysis) for serum Hg but not for whole blood Hg concentrations [86]. We found no differences in Hg levels in either maternal or cord whole blood between the groups with and without amalgam fillings, but the results of multiple regression analysis showed that the number of dental amalgams, if >3, was a determinant of Hg levels in maternal blood, while there was no influence on Hg in hair, placenta, and cord blood. Previous studies reported a positive association between maternal dental amalgams and inorganic Hg in maternal blood [29], cord blood [29,42], and the placenta [83], or no association between maternal amalgam fillings and Hg levels in cord blood [59]. Our results on the association of Hg levels in maternal and cord serum with the number of maternal amalgams are in line with the fact that inorganic Hg in blood is almost equally distributed between red blood cells and plasma, whereas Hg found in blood and bound mostly to hemoglobin in the red blood cells is approximately 90% in the form of MeHg and originates mainly from the diet, especially seafood [12,47,86,89].

### 4.3. Metallothionein Polymorphisms and Hg Biomarkers

External factors that influence vulnerability to the toxic effects of Hg are gender, age, physiological status, nutritional status, and the intake of other foods or nutrients that might influence the absorption, uptake, distribution, and metabolism of MeHg [28,90]. Genetic polymorphisms in candidate genes may explain variations in biomarkers of exposure and inter-individual differences in susceptibility to Hg toxicity as they may affect delivery to the target organs or affect the response of the target organs to Hg. Polymorphisms of several classes of genes have been reported to interact with Hg toxicokinetics and toxicodynamics, such as genes that encode glutathione, selenoproteins, and the metallothioneins’ superfamily, and certain main genetic variants have been found to be related to Hg-induced neurotoxicity [36,82,91,92]. It was found that SNPs in MT1 and MT2 may influence Hg concentrations at levels of exposure relevant to the general population [93]. To this end, our study in mother–infant pairs in Croatia showed no significant interrelationship between maternal MT2 concentration, SNP MT2-5A/G (rs28366003), and concentrations of the main toxic metals (Cd, Pb, and Hg) and selected essential elements (zinc (Zn), copper (Cu), and Se) in maternal blood, placenta, and cord blood of non-smokers and smokers, with the exception of the impact on placental iron (Fe) decrement in non-smoking postpartum women, as reported in our previous paper [44]. In the part of the study presented here, focusing on Hg exposure related mainly to fish intake and amalgam fillings as an additional Hg exposure source, we also found no interrelationship between Hg or Se concentrations in the measured matrices of maternal and fetal origin and maternal SNP MT2-5A/G (rs28366003).

We are well aware that both our research approach (cross-sectional study in mother–infant pairs) and the study sample size (less than 300 participants) are far too small to draw any firm conclusions on the influence of this particular MT2 polymorphism on Hg levels in the measured biological samples of maternal and fetal origins related to the main sources of Hg exposure, seafood, and dental amalgams. This, together with the broader assessment of MT2 levels in the measured matrices, is recognized as the major weakness of this study.

### 4.4. Risk of Prenatal and Postnatal Hg Exposure

Women of child-bearing age should consume fish since a fish-rich diet is beneficial for the health of both mother and developing offspring, and supplies nutrients including essential elements and omega-3 fatty acids. For example, docosahexaenoic acid (DHA) levels were found to decline during pregnancy and lactation, but the higher the intake of seafood and eicosapentaenoic acid (EPA)/DHA-supplements, the higher the DHA status in the maternal circulation [94]. However, women should choose species with a smaller MeHg content, such as sardines, salmon or oysters, and avoid large predatory fish such as tuna, shark or swordfish that have high MeHg content [56,95]. For decades, there has been concern that MeHg bioaccumulated in fish and consumed by pregnant women could lead to neurodevelopmental disabilities of the offspring, as it is easily transferred through the placenta to the fetus. Several wide-scale studies in populations predominantly consuming seafood including fish, with a focus on prenatal exposure and Hg adverse effects on the developing brain, failed to reach consistent data and firm evidence that lower levels of Hg exposure due to maternal seafood consumption contribute to neurodevelopmental disabilities in the offspring [96,97]. A recent review summarizing the evidence of the effects of low-level MeHg exposure pointed to the possibility of impaired fetal and infant growth among susceptible subgroups, though this requires further investigation [67]. Although seafood is a source of dietary Hg, seafood appeared to explain a relatively small proportion of the variation in whole blood total Hg levels in the ALSPAC birth cohort study population in the UK, suggesting that limiting seafood intake during pregnancy may have a limited impact on prenatal blood Hg levels [98].

The results reported here corroborate previous studies that showed no association between birth weight or birth length and maternal seafood consumption [99,100,101,102] or the number of dental amalgams [87,103]. Two controlled clinical trials examining the health effects of amalgam conducted in children—the New England Children’s Amalgam Trial (NECAT) [104] and the Casa Pia study [105]—reported no evidence of adverse psychosocial outcomes from Hg exposure in children. Those and other data provide a good indication that Hg amalgam treatment is safe for the majority of children and is not associated with a risk of neuropsychological deficits [106]. However, recent studies suggest that the influence of genetic polymorphisms on the degree of individual susceptibility to Hg internal exposure and consequent toxicity in children or adults cannot be ruled out. Most recent expert opinions are that there is no current evidence to preclude the use of either amalgam or alternative materials in dental restorative treatment, and that the choice of material should be based on patient characteristics, including pregnancy. The need for further research is recognized, particularly concerning the evaluation of potential neurotoxicity of Hg from dental amalgam in combination with the influence of genetic polymorphisms on individual susceptibility to Hg toxic effects in youth and adults. Knowledge on alternative dental restorative materials should also be expanded [80,81]. Thus, genetic and epigenetic predispositions under conditions of specific Hg contamination will have an impact on future safe limits for Hg with regard to its environmental toxicity. 

### 4.5. Hg, Se, and Fish Consumption

Concentrations of Hg in commercial fish species, including European pilchard and hake, as commonly consumed species by the Croatian population, are reported to range between 0.017–0.130 mg/kg wet weight of muscle fish tissue [23,107]. According to the threshold level of Hg in seafood of 0.2 mg/kg wet weight tailored for Mediterranean coastal populations [108] and considering a fish portion of 130 g and fish consumption of two times per week, the estimated Hg intake in our study participants was in the range of 0.65–0.80 µg/kg body weight per week during pregnancy (according to the data provided in Table 1). This corresponded to 50–62% of the tolerable weekly intake (TWI) for MeHg of 1.3 µg/kg body weight as set by the EFSA Panel on Contaminants in the Food Chain (CONTAM) [8]. From this, it can be concluded that such Hg exposure from fish consumption does not pose a health risk. On the other hand, seafood, especially fish, is an important source of Se, an ultra-trace bioelement that can act as a growth factor, powerful antioxidant, and anticancer agent and is essential for thyroid hormone homeostasis and immunity, while its deficiency can be associated with metabolic processes leading to pathological conditions and diseases [19,20]. Our preliminary results showed that marine fish, especially small pelagic (oily) species, are a valuable dietary source of Se and other essential elements, including Fe, Zn and Cu, while also having low Hg concentrations [109,110]. A favorable Se:Hg molar ratio in fish can balance the risk and benefits related to fish consumption [20,111,112]. We found Se levels of 0.66 to 1.1 mg/kg wet weight in European pilchard from the Adriatic Sea [109] and that Se intake in one 130 g portion of this species contributes more than 122% of the dietary reference value (DRV) of Se, which is 70 µg per day during pregnancy [113]. In healthy omnivores, consumption of 3–4 fish servings per week, including during pregnancy, should be sufficient to ensure the essential intake of omega-3 fatty acids and Se [114]; therefore, there is no need for additional Se supplementation, especially in consumers of fish species low in Hg and high in Se.

A number of studies have reported that maternal serum concentrations of Se decrease with the progress of pregnancy due to increased Se transfer to the developing fetus, especially during the third trimester when Se is retained in the fetal liver [53,115,116]. In our study, fish consumption frequency was low to moderate and concentrations of Se in maternal serum were 53.9 µg/L and 59.0 µg/L in the continental and coastal group, respectively, which are in good agreement with reported Se serum levels of 58.02 µg/L in a subgroup of Spanish women in the 24^th^ week of pregnancy who consumed fish one to two times per week [53]. These levels are slightly below the range of 60–140 µg Se/L in serum/plasma generally recognized in the literature published during the last two decades as an adequate or “optimal” Se status for beneficial health effects, including optimization of iodothyronine deiodinases, glutathione peroxidase and selenoprotein P, protection from certain types of cancers, and reduction in total mortality [117]. In our study, Se levels in the coastal area were 1.2-fold higher than in the continental area for all measured biomarkers. The overall Se level in maternal blood was 78.2 μg/L (interquartile range 69.2–86.5 μg/L), which is in agreement with previous studies in Sweden [42,118,119] and Slovenia [120,121], while lower levels were reported in Italy [52], Canada [122], and the Faroe Islands [22]. Differences in Se levels reported here and in other studies are likely due to the different amounts and species of consumed fish containing different amounts of Se [123,124]. We found a significant correlation between seafood/fish consumption and all Hg and Se biomarkers, as well as between these two elements in most of the measured samples, with the exception of Hg in maternal and cord serum and Se in hair. These results are in agreement with a previous study carried out in Croatian postpartum women that showed correlations between Hg and Se maternal and cord blood levels and fish consumption as a source of intake of both elements [52]. As fish/seafood is the main source of these elements, associations between Hg and Se in different biological samples were also reported by other authors [41,42,83,125], whereas a study in Sweden with high maternal fish consumption reported no such association between serum Se and blood MeHg [72]. In contrast to our findings that Se levels in maternal and cord blood/serum and placenta were positively associated with seafood consumption, a Swedish study reported no association between Se in cord blood and fish consumption, which was explained by the fact that women included in the study had low fish consumption and were taking Se supplements [42]. 

The complex relationship between Se intake, biomarkers of its status in the body, and risk of disease has not yet been fully elucidated. Appropriate biomarkers, together with the impact of gender and genotype, especially polymorphisms, should be studied further and considered when updating the current dietary recommendations to aid preventive public health measures and therapeutic clinical practice [117]. There is also conflicting and inconsistent evidence on the protective role of micronutrients, such as Se in organic compounds (mostly selenomethionine and selenocysteine) in fish eaters regarding the Hg exposure-related neurotoxic effects in humans. Mechanisms for the interaction of Hg and Se were proposed [126,127,128] but are generally not yet fully understood; hence further studies are required [22,58,129,130,131,132].

## 5. Conclusions

The study of mother–infant pair cohorts in continental and coastal areas of Croatia (a country that geographically forms the Mediterranean front of Central Europe), that differ in traditional dietary habits of fish consumption, shows that maternal seafood/fish consumption is associated with increased levels of Hg and Se in maternal hair and blood, umbilical cord blood, and also in placenta, as a tissue sample generally underrepresented as a matrix with determined Hg concentrations. Coastal participants with a cultural tradition of a fish-rich diet were observed to have higher levels of both trace elements, toxic Hg and essential Se. New evidence is related to the lack of association between either maternal serum MT2 and Hg exposure from dental amalgams or MT2A-5A/G (rs28366003) polymorphism and the levels of Hg and Se in the measured matrices. These results confirm that Hg levels in maternal hair and blood are associated with seafood consumption and mainly reflect MeHg exposure, and this is additional evidence of the association of Hg levels in serum and the number of dental amalgams, reflecting inorganic Hg exposure. The results of this study provide new and original data on Hg exposure in the vulnerable population groups of mother–infant pairs in this part of the world. Future studies of Hg and Se effects and their interactions related to fish consumption should include a detailed seafood consumption questionnaire and collection of detailed data on intake frequency, serving sizes, and consumed fish species, together with a larger number of study participants (at least several hundreds or thousands) to assess the impact of the related gene polymorphism.

## Figures and Tables

**Table 1:** General characteristics of the participants from Croatia included in the study (*n* = 281).

		Continental Area *n* = 185		Coastal Area *n* = 96
**Maternal data**				
Age, years		29.4 ± 4.64		29.3 ± 4.76
Education, *n* (%)				
Primary school		11 (6)		6 (6)
Secondary school		124 (67)		64 (67)
University degree		50 (27)		26 (27)
Parity		2 (1–5)		2 (1–5)
Body weight before pregnancy, kg		64.4 ± 11.47		64.3 ± 11.62
Body height, cm		168 ± 6.1		169 ± 5.7
Weight gain during pregnancy, kg		15.3 ± 4.85		14.9 ± 4.73
Gestation week of delivery, week		39.4 ± 1.32		39.6 ± 1.30
**Newborn and placental data**				
Boys, *n* (%)		97 (52)		39 (41)
Girls, *n* (%)		88 (48)		57 (59)
Birth weight, g		3515 ± 442		3452 ± 433
Birth length, cm		51.4 ± 2.15		51.1 ± 1.91
APGAR 1^st^ min, score		10 (7–10)		10 (8–10)
APGAR 5^th^ min, score		10 (8–10)		10 (8–10)
Trimmed placental weight, g		296 ± 58.2		291 ± 57.8
**Seafood (fish) consumption per week, *n* (%)**				
no consumption		20 (11)		4 (4)
≤1		101 (54)		40 (42)
>1–2		50 (27)		31 (32)
>2		14 (8)		21 (22)
**Frequency of *MT2A*-5A/G polymorphism, n (%)**				
Wild-type (AA genotype)			253 (94.4)	
G allele carriers (AG/GG genotype)			15 (5.6)	
**Number of dental amalgams, n (%)**				
none			38 (18)	
1–2			62 (30)	
3–4			46 (22)	
≥5			62 (30)	

Results are presented as mean ± SD, median (min–max) or number (*n*) and percentage (%). APGAR score = vitality index assigned to every newborn infant at one and five minutes after birth based on a rating of 0, 1, or 2 for each of the five characteristics: A—appearance (skin color), P—pulse (heart rate), G—grimace (reflex irritability), A—activity (muscle tone) and R—respiration (respiratory effort).

**Table 2:** Concentrations of total mercury (Hg) and selenium (Se) in subjects from two geographic areas of residence in Croatia.

	*n*	Continental Area	*n*	Coastal Area
**Hg**				
Maternal hair (mg/kg)	71 ^§^	0.20 (0.11–0.35) 0.26 ± 0.21	95	0.45 (0.22–1.06) * 0.88 ± 1.18
Maternal blood (μg/L)	185	0.96 (0.56–1.64) 1.29 ± 1.27	96	2.39 (1.06–4.71) * 3.54 ± 4.31
Maternal serum (μg/L)	185	0.26 (0.10–0.56) 0.44 ± 0.52	96	0.58 (0.31–0.94) * 0.78 ± 0.75
Placenta (μg/kg wet wt)	184	2.49 (1.34–4.48) 3.44 ± 3.40	92	7.00 (2.84–11.3) * 9.49 ± 11.2
Cord blood (μg/L)	184	1.06 (0.55–1.88) 1.64 ± 1.79	96	3.74 (1.27–7.89) * 5.85 ± 7.92
Cord serum (μg/L)	183	0.26 (0.16–0.44) 0.35 ± 0.27	96	0.40 (0.22–0.76) * 0.61 ± 0.66
**Se**				
Maternal hair (mg/kg)	71 ^§^	0.33 (0.30–0.41) 0.35 ± 0.08	95	0.39 (0.33–0.47) * 0.41 ± 0.11
Maternal blood (μg/L)	185	74.4 (68.2–84.1) 75.1 ± 15.1	96	83.8(74.7–95.5) * 85.8 ± 17.2
Maternal serum (μg/L)	185	54.9 (46.5–62.3) 53.9 ± 13.1	96	57.0 (49.4–67.6) * 59.0 ± 11.8
Placenta (μg/kg wet wt)	184	166 (144–191) 178 ± 54	92	192 (179–208) * 195 ± 22
Cord blood (μg/L)	184	73.0 (66.6–80.9) 73.5 ± 13.1	96	82.4 (75.8–93.1) * 85.0 ± 13.1
Cord serum (μg/L)	183	39.4 (35.4–43.9) 39.7 ± 6.7	96	42.4 (37.9–46.7) * 42.3 ± 6.3

Results are presented as median (25–75% interquartile range) and mean ± SD. *n* = number of measured samples (^§^ previously used for other analysis). * Statistical differences (*p* < 0.05) between the study groups tested by Mann–Whitney U test.

**Table 3:** Concentrations of total mercury (Hg) in maternal hair and serum, placenta and cord serum related to the presence of dental amalgam fillings.

	*N*	Without Dental Amalgams	*n*	With Dental Amalgams
**Hg**				
Maternal hair (mg/kg)	30	0.30 (0.17–0.68) 0.47 ± 0.52	106	0.35 (0.18–0.83) 0.76 ± 1.13
Maternal blood (μg/L)	38	1.48 (0.68–2.82) 1.92 ± 1.63	170	1.43 (0.77–2.95) 2.52 ± 3.53
Maternal serum (μg/L)	38	0.32 (0.16–0.50) 0.41 ± 0.34	170	0.44 (0.23–0.78) * 0.65 ± 0.68
Placenta (μg/kg wet wt)	36	3.08 (1.99–6.84) 4.90 ± 4.33	168	3.99 (1.98–8.18) 6.78 ± 9.11
Cord blood (μg/L)	38	1.76 (0.64–4.28) 2.79 ± 2.66	169	1.60 (0.82–4.75) 3.95 ± 6.41
Cord serum (μg/L)	38	0.24 (0.125–0.39) 0.32 ± 0.25	169	0.36 (0.20–0.62) * 0.52 ± 0.55

Results are presented as median (25–75% interquartile range) and mean ± SD. *n* = number of measured samples. * Statistical differences (*p* < 0.05) tested by Mann–Whitney U test.

**Table 4:** Spearman’s correlation coefficients (ρ_s_) for relationships between the frequency of maternal seafood/fish consumption, number of dental amalgams, and biomarkers of mercury (Hg) and selenium (Se) determined in the studied mother–infant pairs from Croatia (*n* = 94–281) *.

Parameter	Seafood	Amalgams	MT2	Hg-H	HgB-M	HgB-UC	Hg-PL	HgS-M	HgS-UC	Se-H	SeB-M	SeB-UC	Se-PL	SeS-M	SeS-UC
**Maternal age**	**0.163**	−0.067	0.053	0.132	**0.202**	**0.179**	**0.121**	**0.151**	**0.185**	−0.068	**0.134**	0.073	0.059	**0.123**	0.087
**Seafood**		−0.147	−0.067	**0.535**	**0.501**	**0.527**	**0.415**	**0.326**	**0.391**	0.122	**0.276**	**0.291**	**0.270**	**0.137**	**0.159**
**Amalgams**			−0.109	0.094	0.041	−0.037	0.030	**0.161**	**0.162**	−0.097	−0.018	−0.052	−0.039	−0.019	−0.002
**MT2**				−0.116	0.025	−0.060	−0.066	0.030	−0.065	0.033	**−0.276**	−0.192	−0.026	−0.076	−0.076
**Hg-H**					**0.820**	**0.853**	**0.800**	**0.625**	**0.761**	**0.270**	**0.375**	**0.425**	**0.271**	**0.221**	**0.229**
**HgB-M**						**0.844**	**0.856**	**0.822**	**0.783**	**0.228**	**0.485**	**0.423**	**0.277**	**0.292**	**0.289**
**HgB-UC**							**0.832**	**0.679**	**0.774**	**0.211**	**0.490**	**0.518**	**0.257**	**0.300**	**0.364**
**Hg-PL**								**0.783**	**0.783**	**0.218**	**0.431**	**0.454**	**0.255**	**0.269**	**0.268**
**HgS-M**									**0.778**	0.121	**0.453**	**0.385**	**0.187**	**0.313**	**0.293**
**HgS-UC**										0.114	**0.410**	**0.419**	**0.214**	**0.238**	**0.341**
**Se-H**											**0.276**	**0.206**	**0.288**	**0.286**	0.051
**SeB-M**												**0.523**	**0.266**	**0.796**	**0.264**
**SeB-UC**													**0.364**	**0.306**	**0.550**
**Se-PL**														**0.183**	**0.202**
**SeS-M**															**0.199**

H—hair; B—blood; S—serum; M—maternal; PL—placenta; UC—umbilical cord. Significant correlations (*p* < 0.05) are marked in bold. * *n* depends on the number of available data: *n* (MT2) = 94, *n* for other variables are shown in Table 1 and Table 2.

**Table 5:** Stepwise multiple linear regression results for relationship of Hg concentration in maternal hair (*n* = 135), blood and serum, and umbilical cord blood and serum (*n* = 205).

Dependent Variable	Equation (Respects the Sequence of Variables Entered in the Equation)	R^2^	*p*
ln(Hg-H)	= 0.51 Seafood consumption + 0.83 Residence area + 0.03 Weight gain + 0.04 Age – 0.19 Parity + 2.33	0.45	<0.0001
ln(HgB-M)	= 0.46 Seafood consumption + 0.77 Residence area + 0.40 Dental amalgams (≥5) + 0.16 Dental amalgams (3–4) – 1.5	0.35	<0.0001
ln(HgB-UC)	= 0.61 Seafood consumption + 0.75 Residence area + 0.16 Education + 0.03 Weight gain – 1.95	0.39	<0.0001
ln(Hg-PL)	= 0.38 Seafood consumption + 0.71 Residence area + 0.03 Weight gain + 0.15 Education – 0.86	0.31	<0.0001
ln(HgS-M)	= 0.77 Residence area + 0.22 Seafood consumption + 0.84 Dental amalgams (≥5) + 0.42 Education + 0.56 Dental amalgams (3–4) + 0.47 Dental amalgams (1–2) – 3.04	0.29	<0.0001
ln(HgS-UC)	= 0.37 Seafood consumption + 0.72 Dental amalgams (≥5) + 0.35 Residence area + 0.51 Dental amalgams (1–2) + 0.51 Dental amalgams (3–4) – 2.55	0.28	<0.0001

Regression model includes the following variables: residence area (1 = continental, 2 = coastal), maternal age (years), education (1 = primary school, 2 = secondary school, 3 = university degree), parity, weight gain during pregnancy (kg), frequency of seafood consumption per week, maternal dental amalgams (transformed to dichotomous variables using zero amalgam fillings as the reference category). Only variables at *p* < 0.05 are included in the final model. H—hair; B—blood; S—serum; M—maternal; PL—placenta; UC—umbilical cord.

## Supplementary Materials

The following are available online at https://www.mdpi.com/2218-273X/10/6/821/s1, Table S1: Temperature program of microwave digestion system UltraCLAVE IV (Milestone, Sorisole, Italy), Table S2: ICP-MS Agilent 7500cx (Agilent Technologies, Tokyo, Japan) optimized working conditions, Table S3: Comparison between the reference values and the obtained values of Hg and Se in the reference materials used.
