# Supplementary material for: Mercury Exposure Assessment in Mother–Infant Pairs from Continental and Coastal Croatia
Source: Biomolecules. 2020 May 27;10(6):821. doi: 10.3390/biom10060821 (PMC7355586; doi:10.3390/biom10060821)
Supplement: Supplementary file 1 [file biomolecules-10-00821-s001.pdf]

Supplementary materials

Journal: Biomolecules

Special Issue "Toxic and Essential Metals in Human Health and Disease"

Title: Mercury Exposure Assessment in Mother-Infant Pairs from Continental and Coastal Croatia

Authors: Ankica Sekovanić, Martina Piasek, Tatjana Orct, Antonija Sulimanec Grgec, Marijana Matek Sarić, Sandra Stasenko, Jasna Jurasović

Corresponding author:

Martina Piasek, MD, PhD, Senior Researcher

Institute for Medical Research and Occupational Health
Ksaverska cesta 2, HR-10000 Zagreb, Croatia

e-mail: mpiasek@imi.hr

Supplementary material contains: 3 Tables

Table S1

Temperature program used for digestion of the hair and placental tissue samples by microwave digestion system UltraCLAVE IV (Milestone, Italy).

	**T (min:s)**	**E (W)**	**T (°C)**	**P (bar)**
1.	5	1000	80	100
2.	10	500	130	100
3.	4:30	1000	180	120
4.	6:30	1000	220	130
5.	20	1000	220	130

Table S2

Operating conditions of ICP-MS Agilent 7500cx (Agilent Technologies, Japan).

**Parameter**		
RF Power	1550 W	
RF matching	1.72 V	
Sampling depth	8.3 mm	
Torch-H	0.9 mm	
Torch-V	-0.1 mm	
Nebulizer pump	0.08 rps	
Plasma gas flow rate	15 L/min	
Makeup gas flow rate	0.13 L/min	
Carrier gas flow rate	1.05 L/min	
Nebulizer	MicroMist (quartz)	
Spray chamber	Scott type (quartz), cooled at 2°C	
Sample cone	Nickel, 1 mm orifice diameter	
Skimmer cone	Nickel, 0.4 mm orifice diameter	
Doubly-charged ions and oxides limits	$^{140}Ce^{2+}/^{140}Ce^{+}$<1.5 %; $^{140}Ce^{16}O^{+}/^{140}Ce^{+}$<1.5 %	
	**no gas**	**H_2**
Collision/reaction gas flow rate	/	3.6 mL/min
Extract lens 1 voltage	0 V	1.5 V
Extract lens 2 voltage	-129 V	-129 V
Isotopes measured	^{202}Hg	^{78}Se

Table S3

Reference values and concentrations of Hg and Se obtained by ICP-MS in analysed reference materials.

	**ClinChek® Whole Blood Control**						**ClinChek® Serum Control**			
**Element**	Level I		Level II		Level III		Level I		Level II	
	Certified value (control range)	Observed value (mean ± SD)	Certified value (control range)	Observed value (mean ± SD)	Certified value (control range)	Observed value (mean ± SD)	Certified value (control range)	Observed value (mean ± SD)	Certified value (control range)	Observed value (mean ± SD)
Hg (µg/L)	1.2 (0.840-1.56)	1.1±0.02	3.04 (2.28-3.80)	3.33±0.07	6.23 (4.98-7.48)	6.70±0.04	1.98 (1.39-2.57)	1.92±0.04	11.0 (8.8-13.2)	10.6±0.2
Se (µg/L)	72.1 (57.7-86.5)	66.4±2.3	111 (88.8-133)	100±5.0	134 (107-161)	118±1.0	83.3 (62.5-104)	76.3±1.4	129 (103-155)	123±9

	**Seronorm™Trace Elements Whole Blood**				**Seronorm™Trace Elements Serum**			
	Level I		Level II		Level I		Level II	
	Analytical value (acceptable range)	Observed value (mean ± SD)	Analytical value (acceptable range)	Observed value (mean ± SD)	Analytical value (acceptable range)	Observed value (mean ± SD)	Analytical value (acceptable range)	Observed value (mean ± SD)
Hg (µg/L)	1.5 (0.90-2.10)	1.6±0.03	16.0 (9.6-22.4)	15.5±0.3	0.62 (0.52-0.7)	0.78±0.06	1.87 (1.57-2.17)	1.90±0.03
Se (µg/L)	59 (35-83)	55±1.0	112 (66-158)	108±2.0	107 (93-121)	97±0.7	163 (143-183)	150±0.2

	**Bovine Liver BCR® 185R**		**Pig Kidney BCR® 186R**		**Mussel Tissue BCR® 278R**	
	Certified value (95% confidence intervals)	Observed value (mean ± SD)	Certified value (95% confidence intervals)	Observed value (mean ± SD)	Certified value (95% confidence intervals)	Observed value (mean ± SD)
Hg (mg/kg)	(0.004-0.007)	0.0049±0.0001	1.97 (0.04)	1.97±0.07	0.196 (0.009)	0.196±0.005
Se (mg/kg)	1.68 (0.14)	1.73±0.017	10.3 (0.5)	10.4±0.1	1.84 (0.10)	1.81±0.04

	**Human Hair IAEA-086**		**Human Hair NIES CRM No.13**	
	Recommended value (95 % confidence intervals)	Observed value (mean ± SD)	Certified value (uncertainty)	Observed value (mean ± SD)
Hg (mg/kg)	0.573 (0.534-0.612)	0.503±0.002	4.42 (0.20)	4.42±0.02
Se (mg/kg)	1.0 (0.80-1.2)	0.92±0.05	1.79 (0.17)	1.67±0.01
